# Drinking beer, wine or spirits – does it matter for inequalities in alcohol-related hospital admission? A record-linked longitudinal study in Wales

**DOI:** 10.1186/s12889-019-8015-3

**Published:** 2019-12-09

**Authors:** Andrea Gartner, Laszlo Trefan, Simon Moore, Ashley Akbari, Shantini Paranjothy, Daniel Farewell

**Affiliations:** 1 Division of Population Medicine, School of Medicine, Cardiff University, 3rd Floor, Neuadd Meirionnydd, Heath Park, Cardiff, CF14 4YS UK; 2 Violence Research Group, School of Dentistry, Cardiff University, Cardiff, CF14 4XY UK; 3 Crime and Security Research Institute, Cardiff University, Cardiff, CF10 3AE UK; 4 Health Data Research UK, Swansea University, Swansea, UK

**Keywords:** Alcohol, Deprivation, Inequalities, Beverage type, Record linked, Hospital admission

## Abstract

**Background:**

Alcohol-related harm has been found to be higher in disadvantaged groups, despite similar alcohol consumption to advantaged groups. This is known as the alcohol harm paradox. Beverage type is reportedly socioeconomically patterned but has not been included in longitudinal studies investigating record-linked alcohol consumption and harm. We aimed to investigate whether and to what extent consumption by beverage type, BMI, smoking and other factors explain inequalities in alcohol-related harm.

**Methods:**

11,038 respondents to the Welsh Health Survey answered questions on their health and lifestyle. Responses were record-linked to wholly attributable alcohol-related hospital admissions (ARHA) eight years before the survey month and until the end of 2016 within the Secure Anonymised Information Linkage (SAIL) Databank. We used survival analysis, specifically multi-level and multi-failure Cox mixed effects models, to calculate the hazard ratios of ARHA. In adjusted models we included the number of units consumed by beverage type and other factors, censoring for death or moving out of Wales.

**Results:**

People living in more deprived areas had a higher risk of admission (HR 1.75; 95% CI 1.23–2.48) compared to less deprived. Adjustment for the number of units by type of alcohol consumed only reduced the risk of ARHA for more deprived areas by 4% (HR 1.72; 95% CI 1.21–2.44), whilst adding smoking and BMI reduced these inequalities by 35.7% (HR 1.48; 95% CI 1.01–2.17). These social patterns were similar for individual-level social class, employment, housing tenure and highest qualification. Inequalities were further reduced by including either health status (16.6%) or mental health condition (5%). Unit increases of spirits drunk were positively associated with increasing risk of ARHA (HR 1.06; 95% CI 1.01–1.12), higher than for other drink types.

**Conclusions:**

Although consumption by beverage type was socioeconomically patterned, it did not help explain inequalities in alcohol-related harm. Smoking and BMI explained around a third of inequalities, but lower socioeconomic groups had a persistently higher risk of (multiple) ARHA. Comorbidities also explained a further proportion of inequalities and need further investigation, including the contribution of specific conditions. The increased harms from consumption of stronger alcoholic beverages may inform public health policy.

## Background

Alcohol consumption is a leading risk factor for population health worldwide [1]. Measures of alcohol-related harm such as hospital admissions and mortality show particularly wide inequalities and reducing inequalities is a focus of governments [1–4]. Alcohol-related harm has been found to be higher in disadvantaged groups, despite comparable or even lower reported alcohol consumption than in advantaged groups [5, 6]. This phenomenon has been termed the ‘alcohol harm paradox’. A number of hypotheses to explain it have been suggested in the literature [5, 7–9].

The first hypothesis is that there may be different patterns of alcohol consumption across groups rather than simply unit consumption or whether a threshold of consumption is reached. Overall, average consumption may not differ between groups but if all alcohol is consumed in one sitting peak toxicity is greater in those who binge drink. More deprived groups are more likely to drink at extreme levels, potentially in part explaining the paradox [8]. The type of alcoholic beverage may also offer an explanation. Consumption of spirits or beer has been associated with worse “trouble per litre” than wine, and consumption of spirits have been associated with increased alcohol poisoning and aggressive behaviour [10, 11]. It has also been suggested that the poorest outcomes are found for beverages chosen by young men [10]. A potential mechanism could be the faster absorption of alcohol from stronger drinks or other characteristics of the people with a particular beverage preference, but the reasons for differing outcomes by beverage type are not well understood.

The second hypothesis concerns the combination of challenging health behaviours or comorbidities typically found in more disadvantaged groups. This combination causes proportionately poorer outcomes compared to similar alcohol consumption in advantaged groups. Deprived higher risk drinkers were found to be more likely to drink alcohol combined with other “health-challenging behaviours that include smoking, being overweight, poor diet and lack of exercise” compared to more affluent groups [7]. There are also known associations between mental health and alcohol consumption which could affect disadvantaged groups differently [12].

The third hypothesis relates to underestimating consumption in disadvantaged groups and the alcohol harm paradox not existing or being an artificial construct. Response bias may be at work where those who do not respond to the survey could have systematically different consumption levels or worse outcomes compared to responders [13]. Moreover, current drinking may not reflect the life history of harmful drinking, which has been found to be associated with deprivation in lower and increased risk drinkers [7].

A few recent cross-sectional studies have investigated the harm paradox, but mostly considered drinking patterns and their influence on the paradox rather than outcomes of harm [7, 8]. Only one longitudinal study in Scotland has employed record-linkage between consumption patterns and harm, investigating socioeconomic status as an effect modifier, but did not include the type of beverage or multiple admissions [5].

This study aims to investigate whether and to what extent individual alcohol consumption by type of beverage, smoking, BMI and other factors could account for inequalities in alcohol-related hospital admission (ARHA). A different risk of harm by socioeconomic group for a given level of individual consumption could be an explanation of the alcohol-harm paradox at group level. Additionally, we examine how the patterns of consumption by type of beverage differ by socioeconomic group.

## Methods

### Data

This analysis was carried out using the Electronic Longitudinal Alcohol Study in Communities (ELAStiC) data platform and details on the data and linkage methods are outlined in the study protocol [14]. A summary and further specific details for this study are described below.

### Welsh health survey

Our cohort consisted of 11,038 people aged 16 and over who responded to the Welsh Health Survey in 2013 and 2014, consenting to have their survey responses linked to routine health data. The Welsh Health Survey is an annual population survey on health and health-related lifestyle based on a representative sample of people living in private households in Wales (random sampling). It consists of a short interview with the head of household and a self-completed questionnaire for each individual adult aged 16 years and above in the household. A question on consent for data linkage was included from April 2013 to December 2014 and approximately half of the respondents agreed. Originally 11,694 respondents agreed to their data being linked, and records were successfully linked and anonymised into the SAIL Databank through standard split file processes for 11,320 individuals (3.2% loss) [14]. Linkage to records of household residence needed for analysis failed for 282 respondents, resulting in the final sample of 11,038 people (5.6% loss overall). An overview of characteristics of the study population is shown in Table 1.

**Table 1:** Characteristics of the study population

	Men	Women	Total
Survey year			
2013	1906 (37%)	2269 (38%)	4175
2014	3199 (63%)	3664 (62%)	6863
Age group			
16–29 years	716 (14%)	998 (17%)	1714
30–44 years	914 (18%)	1202 (20%)	2116
45–59 years	1277 (25%)	1522 (26%)	2799
60–74 years	1518 (30%)	1502 (25%)	3020
75+ years	680 (13%)	709 (12%)	1389
Area deprivation			
More deprived 40%	1826 (36%)	2170 (37%)	3996
Less deprived 60%	3279 (64%)	3763 (63%)	7042
Alcohol consumption*			
None	526 (10%)	854 (13%)	1380
Not binge	3041 (64%)	3740 (69%)	6783
Binge	1440 (26%)	1197 (18%)	2637
Mean units (drinkers only)			
Beer or Cider	6.3 (6.7)	1.6 (3.7)	4.0 (6.1)
Wine or Champagne	2.1 (4.1)	3.8 (4.7)	2.9 (4.5)
Spirits or other	1 (2.7)	1.5 (3.1)	1.2 (2.9)
Any type	9.5 (7.8)	6.9 (5.8)	8.2 (7.0)
Smoking status*			
Never smoker	2242 (44%)	3073 (52%)	5315
Ex-smoker	1837 (36%)	1670 (28%)	3507
Smoker	972 (19%)	1136 (19%)	2108
Mean BMI (SD)	27.2 (4.84)	27 (5.94)	27.1 (5.4)
Total person-years	29,221.1	34,417.8	63,638.9
Number of admissions	169	110	279

Number of respondents (%) or mean units (Standard deviation, SD)

*Numbers do not sum due to missing data

### Measures of socioeconomic status

We used an area-based deprivation measure (i), the Welsh Index of Multiple Deprivation (WIMD) 2011 [15], as well as four individual-level measures of socioeconomic status from survey responses (ii) social class, iii) employment, iv) housing tenure, and v) highest qualification). We linked the WIMD to each Lower layer Super Output Area (LSOA) of residence at survey month. We grouped the two more deprived quintiles and three less deprived quintiles because of relatively small numbers.

### Alcohol consumption

Respondents were also asked about the frequency of drinking, including whether or not they had drunk alcohol at all during the past year and the number of each type of alcoholic beverage they had consumed on the heaviest drinking day in the past week. These include categories of, for example, “small can of strong beer”, “small glass of wine”, as well as free text for additional drinks not listed. These data were converted into units (8 g ethanol per unit) consumed by beverage type, and capped at 60 units to deal with a very small number of responses of between 60 and 120 units, likely a misreading of units. We created three groups: 1) beer and cider; 2) wine and champagne; 3) spirits, alcopops, fortified wine and others. There were relatively small numbers of alcopops, fortified wine and others and so we combined these with the spirits. Our sensitivity analysis showed that the inclusion of these drinks did not alter the results for this category which was predominantly made up of spirits.

### Outcome measure of alcohol-related hospital admission

The outcome was (multiple) alcohol-related hospital admission(s). We selected the earliest episode in each hospital spell with a wholly attributable diagnosis included in the definition outlined in the study protocol [14]. These are similar to the alcohol-specific definition used by Public Health England with a few additional codes [14, 16]. These could be the primary diagnosis or a secondary diagnosis in any position. This included multiple admissions for survey respondents. The details of the data source, linkage and extraction are outlined in the study protocol [14].

### Other survey measures

Other measures used based on survey responses were smoking, BMI, general health and being treated for a mental health condition. Smoking was coded into three categories: 1) regular or current smoker, 2) Ex-smoker and 3) never smoker. BMI was readily calculated based on self-reported height and weight. Respondents were asked about their general health which we coded into the following two groups: 1) Poor and fair health, 2) good, very good and excellent health. Respondents were also asked whether they were currently being treated for depression, anxiety or another mental illness (yes/no). This was coded into a binary variable with values of being treated for any mental health condition listed or not treated if none was selected.

### Study design/processing

**Fig. 1:**
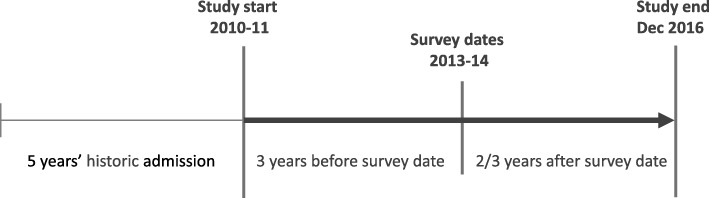
Illustration of study timeline

Survey responses were record-linked within the SAIL Databank to hospital admission data (Patient Episode Database for Wales), mortality data (Annual District Death Extract from the Office for National Statistics) and data containing residence and thus house moves (Welsh Demographic Service Dataset) as outlined in the study protocol [14]. All data was extracted for eight years before the survey month until the end of the year 2016. The study period ran from three years before the survey in 2013 or 2014 to the end of 2016, with a study period of between five and six years depending on when the survey was undertaken. We structured the data so that each person could contribute multiple time periods, if they had an admission, with the number of admissions up to the current time period counted during the study. We also considered the number of historic alcohol-related admissions during the five years before study start (i.e. 8 years before to 3 years before the survey date, or 2005–06 to 2010–11) as a covariate in the modelling analysis. We censored for death or moving out of the study area (Wales). An illustration of the study timeline is shown in Fig. 1. We also performed a sensitivity analysis using the data restricted to time periods after the survey date only (2013/14 to the end of 2016) for comparison.

### Statistical analyses

We estimated hazard ratios (HR) with 95% confidence intervals (95% CIs) for the risk of (multiple) alcohol-related hospital admission associated with each socioeconomic group using multi-level Cox mixed effects models [17]. We used a recurrent event model with admission as the outcome and using age as the underlying timescale rather than calendar time. We used Cox proportional hazards models stratified by the current count of admission events to date (during the study period), so that each unique admission count has a separate baseline hazard function. Including admission counts during the study period as strata accounts for covariance within an individual’s recurrent events and is similar to a frailty model [18]. Details of covariates in each model are given below, but in every case their hazard ratios were assumed constant across strata. Additionally, a random effect at the household level was used in the multilevel analysis to allow for potential similarities in responses within a household over and above their individual characteristics. All analyses were conducted using R [20], specifically using the coxme function [21]. To deal with missing observations for BMI, unit consumption, smoking and individual-level socioeconomic measure we used 20 iterations of multiple imputation using chained equations using the package MICE in R [19]. This was chosen for efficiency to avoid reducing the sample size.

The number of historic events during the 5 years before study start was included as a covariate in all models. This was chosen to account for differences in risk of the next admission, because people with a prior admission were more likely to have another admission than those who did not.

The first basic model (Model A) adjusted for area deprivation, sex and the number of historic ARHA during 5 years before study start. Model B additionally adjusted for the number of units reported by drink type (beer and cider; wine and champagne; spirits including alcopops) on the heaviest drinking day in the past week, smoking status and BMI. We repeated the basic and adjusted model using area deprivation (i) for all other individual measures of socioeconomic status, ii) social class, iii) employment, iv) housing tenure, and v) highest qualification, to compare estimates in the basic model with those of the adjusted model. We also included an interaction term in adjusted Model B between BMI and total unit consumption.

Model C, also based on the adjusted model B, additionally included self-reported general health, and Model D added self-reported treatment for a mental health condition to investigate comorbidities.

Two additional models were used to investigate the contribution of the units for each specific beverage type to inequalities. These were based on Model A, but also included the total units consumed and, separately, the units for each type of drink as covariates (results not shown). Another model included the frequency of drinking (results not shown).

For the sensitivity analysis we have re-run all models above on the limited dataset including only the time periods following the survey date. The results were compared to the main results using the extended dataset.

**Fig. 2:**
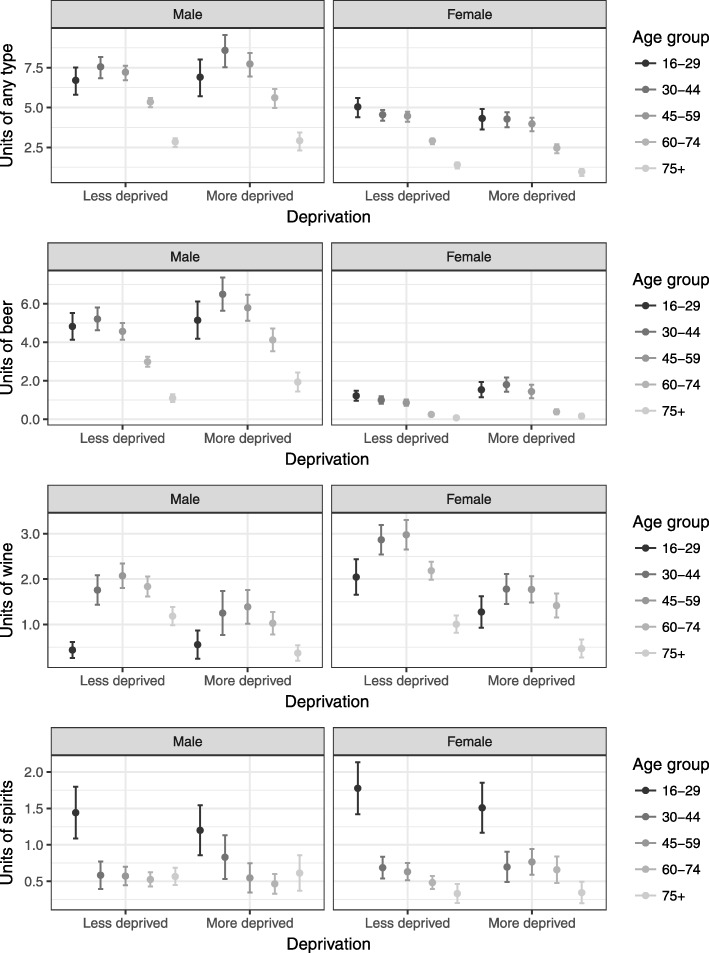
Mean units for by beverage type, age, sex and deprivation group (including 95% confidence intervals)

Finally, we also analysed the mean units of alcohol consumed by beverage type and by age, sex and deprivation group, including 95% confidence intervals (Fig. 2). To show the distribution of units in each group we have also included boxplots for any type of beverage with the outliers removed due to data non-disclosure rules associated with the record-linked environment.

## Results

### Sample characteristics

Our study sample consisted of 11,038 respondents with a total of 63,638.9 person-years of follow-up. There were 279 alcohol-related admissions during the study period (131 individuals with one or more admission). The crude rate per 1000 person-years was 4.38. An overview of our sample characteristics is shown in Table 1 . There were more females than males. Key demographic data was complete in the survey but there were missing responses to some of the individual survey questions, ranging from 0.6% for drinking frequency to 4.9% for BMI. Modelling analyses use imputation to deal with missing responses, but Table 1 shows completed and valid responses only and therefore the sums for each characteristic may be different, for example between sums for alcohol consumption and smoking status.

### Patterns of consumption

Deprived groups had larger proportions of people who reported not drinking at all in the past year (15% compared to 11%, Table 2), and also higher proportions who did not drink in the past week but reported some drinking in the past year (47% compared to 37%, Table 2). However, those who drank in the deprived group had slightly higher proportions of people who binged (more than 4 units for men and more than 3 units for women) on a single occasion, with 25.8% in the deprived group compared to 23.6% in the less deprived group. This suggests that fewer people drank in deprived groups but, those who had any alcohol, drank more. Some of those who either did not drink at all in the past year, or reported some drinking in the past year but no units in the past week had an alcohol-related admission at some point during the study period. This could suggest that ongoing health concerns might explain their abstinence [22].

**Table 2:** Alcohol consumption by deprivation group and whether admitted

	Less deprived		More deprived	
	N (%)	with admission	N (%)	with admission
Drinking frequency*				
Not at all in the past year	785 (11.2)	8	595 (15.0)	7
Less than weekly	2404 (34.3)	9	1575 (39.7)	19
More than weekly	3813 (54.5)	43	1802 (45.4)	45
Binge drinking*				
None in past week	2573 (37.3)	17	1834 (46.9)	28
Some but not binge	2688 (39.0)	19	1068 (27.3)	16
Binge	1628 (23.6)	22	1009 (25.8)	27
Drank at least one unit of*				
Beer or Cider	2121 (30.8)	27	1269 (32.4)	31
Wine or Champagne	2336 (33.9)	16	759 (19.4)	13
Spirits or other	1242 (18.0)	14	656 (16.8)	16
Total cohort	7042	60	3996	71

*Numbers do not sum due to missing data

Overall, the mean units of total alcohol consumed were similar or slightly higher in the more deprived group than the less deprived group for males but similar or slightly lower for females (Fig. 2). If only those who drank are compared (chart not shown) then men in the more deprived group drank more on average than men in the less deprived group for all age groups with smaller differences in women.

**Fig. 3:**
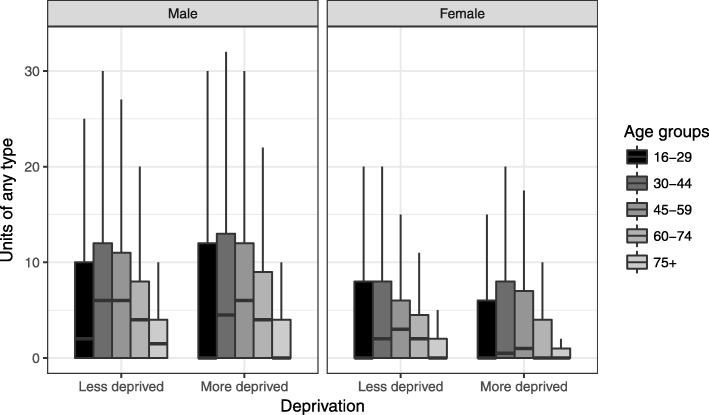
Box plot for any type of beverage by age group, sex and deprivation group (outliers removed)

Socioeconomic patterns differed by type of beverage. Similar to any type, mean units of beer were slightly higher in more deprived groups, and unit consumption much higher for men than women. The pattern for wine was the opposite showing lower consumption in more deprived, with the exception of the youngest men. More spirits were consumed by younger drinkers with only slightly lower averages for the deprived group. There was little difference in the more deprived group in most other age groups of those aged 30 and above compared to less deprived groups. The box plots in Fig. 3 for units of any type of beverage show that the distribution is skewed towards lower reported units reflecting the large proportion of people reporting zero units, particularly in the youngest and oldest age groups. The medians for younger males in more deprived groups are lower than the less deprived, and for females the medians are lower in the more deprived for most age groups.

### Factors associated with alcohol-related hospital admission

A total of 131 out of 11,038 respondents had at least one ARHA during the study period. Women tended to have a lower risk of admission than men (HR 0.71; 95% CI 0.51–0.99, Model A in Table 3), although this was only statistically significant in Model A, and not in the fully adjusted Model B. Smoking had the strongest association with alcohol-related hospital admission and smokers were 4.53 times more likely to have an admission (HR 4.53; 95% CI 2. 85–7.21, Model B) than those who were never smokers. Ex-smokers were 1.50 times more likely to have an admission compared to the same reference group, although this was not statistically significant. BMI appeared to be slightly protective, but it was not statistically significant (HR 0.98; 95% CI 0.94–1.01, Model B). We also investigated the interaction between BMI and total unit consumption based on Model B but we found no evidence for an interaction (results not shown).

**Table 3:** Results of regression models using area deprivation: hazard ratios for the risk of alcohol-related hospital admission for each model covariate

	Basic model (Model A)	Adjusted model (Model B), adjusted for units, smoking and BMI
	HR (95% CI; *p*-value)	HR (95% CI; *p*-value)
Men (ref)	1	1
Women	0.71 (0.51–0.99; 0.046)	0.72 (0.50–1.06; 0.095)
Less deprived 60% (ref)	1	1
More deprived 40%	1.75 (1.23–2.48; 0.002)	1.48 (1.01–2.17; 0.043)
Number of historic adm.	1.38 (1.28–1.49; < 0.001)	1.38 (1.26–1.52; < 0.001)
Units beer and cider		1.02 (0.99–1.05; 0.137)
Units wine and champagne		1.03 (0.99–1.07; 0.127)
Units spirits and other		1.06 (1.01–1.12; 0.016)
Never smoker (ref)		1
Ex-smoker		1.50 (0.90–2.49; 0.119)
Smoking		4.53 (2.85–7.21; < 0.001)
BMI		0.98 (0.94–1.01; 0.224)

HR: hazard ratio; 95% CI: 95% confidence interval; *N* = 11,038; 148 events in less deprived, 131 in more deprived

Unit increases of spirits drunk were positively associated with increasing risk of ARHA (HR 1.06; 95% CI 1.01–1.12, Model B), higher than for other drink types. Unit increases for beer and wine were, however, not statistically significant.

The reported frequency of consumption suggested an elevated risk of ARHA for those who did not drink in the past year and those who drank weekly relative to those who drank less than weekly, although not statistically significant (results not shown). An increased risk for those who did not drink at all might suggest that these are ex-drinkers who have stopped drinking perhaps due to poor health. Due to the relatively small sample size we could not analyse ex-drinkers separately.

People with poor health had an elevated risk of ARHA (HR 2.89; 95% CI 1.91–4.37, Model C) compared to those who considered themselves in good health. Similarly, people who were currently being treated for mental illness had a much higher risk of ARHA than those who did not (HR 2.66; 95% CI 1.72–4.11, Model D). Although this will need further research relating to interactions and specific conditions, it does suggest that comorbidities, either relating to alcohol or otherwise, could be important.

The number of historic admissions before study start was significantly associated with a higher risk of ARHA. We treated this not as a “risk factor” itself, but as merely indicative of the likely presence of other (unmeasured) risk factors.

### Inequalities in the risk of alcohol-related hospital admission

People living in more deprived areas had a higher risk of ARHA (HR 1.75; 95% CI 1.23–2.48) compared to less deprived (Table 3). In an interim model adjusting for units of alcohol drunk only (results not shown), there was little change (4%) in the risk of ARHA for more deprived areas (HR 1.72; 95% CI 1.21–2.44). Adjustment for smoking status and BMI in model B reduced the risk of ARHA by 35.7% (HR 1.48; 95% CI 1.01–2.17).

We found a similar pattern for all socioeconomic measures, area-based or individual-level, of a reduced but still persistently higher risk in disadvantaged groups after adjustment (Table 4). For example, using social class, people in the “Routine and manual” class had a higher risk of ARHA (HR 2.03; 95% CI 1.30–3.15) compared to the “Professional and managerial” class. After adjustment in the full model the risk had slightly reduced but is still substantially higher (HR 1.81; 95% CI 1.09–3.00) than the comparison group.

**Table 4:** Comparison of regression model results: hazard ratios for the risk of alcohol-related hospital admission for each socioeconomic measure

	Events	Person-years	Basic model	Adjusted model
			HR (95% CI; *p*-value)	HR (95% CI; *p*-value)
i) Area deprivation (Model A/B)				
Less deprived 60% (ref)	148	39,801.1	1	1
Most deprived 40%	131	23,837.7	1.75 (1.23–2.48; 0.002)	1.48 (1.01–2.17; 0.043)
ii) Social class (NSSEC)				
Professional and managerial (ref)	80	25,623.1	1	1
Intermediate	39	11,277.2	1.52 (0.86–2.7; 0.152)	1.3 (0.67–2.52; 0.436)
Routine and manual	146	25,297.1	2.03 (1.3–3.15; 0.002)	1.81 (1.09–3; 0.022)
Never worked/long-term unempl.	14	1441.5	5.65 (2.49–12.82; < 0.001)	4.04 (1.55–10.51; 0.004)
iii) Employment				
Employed (ref)	36	30,724.8	1	1
Not employed	243	32,914.1	3.87 (2.24–6.69; < 0.001)	3.38 (1.97–5.65; < 0.001)
iv) Housing Tenure				
Home owner (ref)	127	47,376.9	1	1
Private rental	15	7104.5	1.13 (0.56–2.27; 0.729)	1 (0.49–2.07; 0.992)
Social rental	137	9154.4	3.97 (2.73–5.77; < 0.001)	2.89 (1.9–4.42; < 0.001)
v) Highest qualification				
Degree (ref)	66	11,254.9	1	1
Other	117	39,486.6	1.25 (0.72–2.15; 0.428)	1.03 (0.57–1.84; 0.926)
None	96	12,897.4	2.38 (1.32–4.31; 0.004)	1.78 (0.93–3.4; 0.083)

HR: hazard ratio; 95% CI: 95% confidence interval; N = 11,038

Adjusting for the total number of units regardless of type of beverage (results not shown) gave very similar results to Model B with an elevated risk of ARHA in the most deprived group (HR 1.46; 95% CI 1. 01–2.11). This suggests that the type of beverage was not important over and above the number of units relating to inequalities.

For models C and D the risk of ARHA in the more deprived group was reduced further compared to Model B (Poor health by 16.6%: HR 1.36; 95% CI 0.92–2.00; being treated for mental health condition by 5.0%: HR 1.45; 95% CI 0.96–2.17, Table 5). This risk in disadvantaged groups, although still elevated, was not statistically significant. Although this will need further research relating to interactions and specific conditions, it suggests that comorbidities, either relating to alcohol or otherwise, could be important.

**Table 5:** Results of regression models for area deprivation investigating comorbidities: hazard ratios for the risk of alcohol-related hospital admission for each model covariate

	Adjusted model, including general health (Model C)	Adjusted model including treated for mental health condition (Model D)
	HR (95% CI; *p*-value)	HR (95% CI; *p*-value)
Men (ref)	1	1
Women	0.71 (0.47–1.06; 0.092)	0.63 (0.42–0.95; 0.026)
Less deprived 60% (ref)	1	1
More deprived 40%	1.36 (0.92–2.00; 0.120)	1.45 (0.96–2.17; 0.074)
Number of historic adm.	1.35 (1.22–1.48; < 0.001)	1.35 (1.23–1.47; < 0.001)
Units beer and cider	1.03 (1–1.05; 0.052)	1.02 (0.99–1.04; 0.197)
Units wine and champagne	1.03 (1–1.07; 0.068)	1.03 (0.98–1.07; 0.239)
Units spirits and other	1.07 (1.02–1.13; 0.009)	1.06 (1.01–1.12; 0.025)
Never smoker (ref)	1	1
Ex-smoker	1.38 (0.83–2.32; 0.216)	1.48 (0.88–2.51; 0.141)
Smoker	4.10 (2.56–6.56; < 0.001)	3.88 (2.37–6.35; < 0.001)
BMI	0.97 (0.93–1.01; 0.101)	0.98 (0.94–1.02; 0.257)
Good health (ref)	1	
Poor health	2.89 (1.91–4.37; < 0.001)	
Not treated for mental health condition (ref)		1
Treated for mental health condition		2.66 (1.72–4.11; < 0.001)

HR: hazard ratio; 95%CI: 95% confidence intervals; Model C: N = 11,010, 278 events; Model D: *N* = 10,665, 267 events

### Sensitivity analysis using limited dataset following the survey date only

Using the data limited to the time periods following the survey date there were 131 admissions, 60 in the less deprived and 71 in the more deprived group. There were 33,067 person-years of follow-up. The model results and conclusions drawn overall are similar, but due to smaller number of events most results were not statistically significant (Table 6 in Appendix 1). Inequalities based on area deprivation were slightly narrower, and inequalities based on individual-level socioeconomic measures slightly wider before adjustment compared to the main analysis shown in the paper. Adjustment for alcohol consumption by type, smoking and BMI reduced inequalities, and as before a higher risk of ARHA in disadvantaged groups remained. Adjustment resulted in a similar reduction of the hazard ratio in the repeated Model A and Model B for area-deprivation, but due to smaller inequalities yielded a slightly higher percentage reduction than the extended dataset. Adjustment for poor health or mental health also reduced inequalities further. The risk of ARHA by type of drink was also similar, with the highest risk for spirits. The sensitivity analysis showed that the results are comparable to those shown in the paper using the extended dataset. We decided to sacrifice a small amount of bias relating to the timing of the survey in favour of reducing variance and used the extended analysis as the main analysis in this paper.

## Discussion

The main aim was to investigate whether and to what extent adjustment for individual alcohol consumption by type of beverage and other factors could explain inequalities in alcohol-related hospital admissions and therefore help explain the alcohol harm paradox. We found that consumption by beverage type did not help to explain inequalities in alcohol-related harm, despite consumption by type being socioeconomically patterned. Adjustment for individual-level units by type of alcohol drunk only very slightly reduced inequalities in ARHA, similar to all units combined. Smoking and BMI accounted for part of the differences, reducing inequalities by 35.7%, but deprived groups still had a persistently higher risk of ARHA, having considered multiple admissions. This pattern was similar for area-based deprivation or individual-level socioeconomic measures.

Our findings on inequalities are broadly similar to a previous study [5] which found that disadvantaged groups had consistently higher alcohol-attributable outcomes, having considered similar total alcohol consumption, BMI and smoking. They analysed quintiles of deprivation and more subgroups for the individual socioeconomic measures, as well as a slightly different definition and so a precise direct comparison of the extent of inequalities and effect of the adjustment is difficult. Their study design is also different in analysing the time to the first admission whilst excluding those with a prior admission. Our analysis includes multiple hospital admissions during the study period as well as information on historic admissions. We found historic admission to be an important factor for the risk of another admission. Thus, we incorporated people with multiple admissions during the study period, who use more health service resources and their exclusion or censoring after one admission could potentially exclude certain patterns. For example, descriptive statistics issued by government or health services can include the same people in successive time periods in cross-sectional analyses.

Including the type of beverage in our analysis was novel. Unit consumption per type of drink is not usually available in survey data, either record-linked or not. Whilst beverage type was not important relating to inequalities in ARHA, there were differences in the risk of ARHA by type of drink. Spirits had the highest increase of risk of ARHA per unit increase consumed. A Finnish study found that consumption of spirits increased in direct proportion to overall consumption as part of binge drinking sessions although not investigating subsequent alcohol-related harm [11]. They suggested that whilst beer was consumed in large quantities at a variety of drinking occasions, spirits were “needed to get really drunk” [11]. Others have argued that the most harmful drink is “whatever young men are drinking” [10]. In our study, the average spirit consumption is highest in the younger age group, although higher in young women than in men. The mechanism for increased ARHA for spirits needs further attention and could be due to the faster absorption of alcohol from stronger drinks in one binge drinking session or “pre-loading” before going out in younger people. If policy sought to tackle stronger drinks in particular, they may, however, be replaced by other types rather than reducing harmful consumption.

The alcohol harm paradox is based on deprived groups drinking similarly or even less than advantaged groups on average. In our study, average binge drinking was slightly higher in deprived groups than less deprived. The mean units for any type of alcohol, however, were similar or lower in deprived groups for most age groups. There were differences in proportions of non-drinkers between deprivation groups that influence the averages. This might suggest that the alcohol harm paradox could in part be an artificial construct, particularly when relying on binge drinking measures beyond a threshold instead of individual units, related to the third hypothesis. In our modelling analysis we focussed on inequalities given similar consumption, thereby adjusting for slightly higher average consumption in more deprived groups in our sample, and investigating an important part of the alcohol harm paradox. The type of beverage showed different socioeconomic patterns, in line with international findings on “trouble per litre” [10] and a study in England [7]. The deprived group drank more beer (or cider), but less wine compared to less deprived. The average units of spirits were similar in the deprived and less deprived group in those over the age of 30, but slightly lower in deprived younger people. This may support the finding elsewhere that the paradox may be more concentrated in men and younger age groups, as the association between consumption and socioeconomic status increased with age [9]. Whilst there may not be any inherent difference between units by type and resulting harm, choices may be indicative of different drinking occasions such as binge drinking or other individual factors.

In our models we also investigated self-reported health status and, separately, being treated for a mental health condition. Either adjustment reduced inequalities in ARHA further, suggesting that comorbidities may explain some of the alcohol harm paradox. Socioeconomic deprivation has been shown to be associated with multi-morbidity, particularly mental health conditions [23]. These may also include conditions related to smoking, which we have accounted for in our models, and may explain the relatively small effect of comorbidity reducing inequalities in our models. We were restricted by sample size and study design to analyse this in more detail, but further research should investigate comorbidities further, including specific conditions.

As with all longitudinal studies, following people over time yields detailed information about the dynamics of response to exposures. Another key strength of our study is the use of record-linkage of individual-level alcohol consumption and other factors to alcohol-related harm, as well as multiple measures of socioeconomic disadvantage. To our knowledge this is the first longitudinal linkage study on the alcohol harm paradox investigating the type of beverage and considering multiple admissions. It takes full advantage of the richness of the data through multi-level multi-failure modelling, imputation for missing data, and censoring for migration and death. There are, however, some limitations relating to the data.

The main limitation relates to the relatively small study sample of just over 11,000 respondents and the fact that only around half of those asked agreed to data linkage. This meant that the number of events was also relatively small with 279 admissions in 131 individuals, but were reflecting the uncertainty in the models appropriately. Failure of linkage of survey respondents to residence data was small (3.2%). Further details on linkage of this dataset are included in the ELAStiC study protocol [14]. We have compared the demographic characteristics of our sample to the total sample for both years outside of the record-linked environment and found that the distribution by age and sex is fairly similar. The reported binge drinking patterns by age and sex were also found to be similar, although proportions were slightly lower in our sample. Whilst we have been able to compare alcohol consumption in our sample and the total sample, it is possible that the study sample is different in terms of their ARHA and potentially not population representative. Even with higher consent for linkage a Scottish study found that underestimation of consumption in surveys was likely to be socioeconomically patterned, as was linked alcohol-related harm [13]. The available sample size also meant that we needed to group the more deprived 40% and the less deprived 60% rather than analysing deprivation quintiles. This allowed detection of significant effects, but meant that we are underestimating the extent of inequalities between the more extreme ends of the deprivation gradient. However, we were able to repeat the analyses using individual-level socioeconomic measures allowing some validation of the patterns found, and our results were similar to the only other comparable longitudinal study. Using only conditions wholly attributable to alcohol in our analysis is also underestimating the wider alcohol-related harms where alcohol is only in part responsible.

One of the explanations of the alcohol harm paradox relates to the accuracy of the measure of consumption. We had to assume that reported consumption and other factors are constant throughout the study period, estimated from the survey response in the middle of the study period rather than baseline. We acknowledge the possibility that respondents may have changed their drinking or the reporting of their drinking following a hospital admission and thus the possibility of reverse causation. To circumvent this possible source of bias we performed a sensitivity analysis, using data limited to time periods following the survey date only, which showed substantively similar results. We therefore decided to sacrifice a small amount of bias relating to the timing of the survey in favour of reducing variance. In our study we found a small number of respondents who reported not drinking at all in the past year but having an ARHA during the study period. They could be “sick quitters” who may drink less due to excessive alcohol use in the past or ill health, and likely to have different outcomes to other non-drinkers. Our main measure is self-reported unit consumption, including by type of drink, for the heaviest drinking day in the past week. It may be more indicative of binge drinking in one session than overall units consumed, for example following weekly consumption guidelines. Whether at baseline or not, responders may not recall their actual consumption or give favourable estimates, or their drinking in the past week, as is commonly asked in many surveys, is not representative of their usual or overall consumption. There are some respondents who did not drink in the past week or below binge levels but also had an ARHA.

Reducing inequalities in health is a major goal of governments, and included in the United Nations sustainable development goals [24], and the Wellbeing of Future Generations Act in Wales [2]. Alcohol policy aiming to reduce consumption in populations as a whole, including taxation and reducing availability internationally, tends to have a greater effect on poorer drinkers than on richer drinkers, and may help reduce inequalities in alcohol harm [1]. However, it is not clear whether heavy drinkers with the worst outcomes are affected equally. Some have advocated more focus on targeting specific sub-groups such as extreme drinkers living in poverty or long-term unemployed men [8]. The Welsh Government are due to introduce a minimum unit pricing policy in Wales during 2020 [25], which will likely increase the price of very cheap spirits in supermarkets or off-licences, but may not change prices of spirits in bars or pubs greatly. Future research is needed to investigate whether and how alcohol-related harm may change as a result, particularly with respect to inequalities. Our results relating to increased harm from spirits could help inform policy and the development of interventions around promotions of stronger drinks.

## Conclusions

Considering consumption by type of beverage did not help explain inequalities in alcohol-related harm, despite consumption being socioeconomically patterned. Smoking and BMI explained part of these differences, reducing inequalities by 35.7%, but deprived groups still had a persistently higher risk of (multiple) ARHA. Although more people in deprived areas were abstaining from alcohol, those who consumed alcohol drank more heavily. Deprived drinkers drank more beer (or cider) and in most age groups also spirits, but less wine compared to less deprived drinkers. Whilst type of beverage was not important relating to inequalities in ARHA, there were differences in the risk of ARHA by type. One potential mechanism for the increased ARHA for spirits could be the faster absorption of alcohol from stronger drinks in one binge drinking session or “pre-loading” before going out in younger people. Our results could help inform interventions on reducing promotions of stronger drinks. The minimum unit pricing policy due to be implemented in Wales during 2020 will likely increase the price of some spirits in supermarkets and off-licences and our results may inform research evaluating the effect for type of beverage, but also inequalities in alcohol-related harm. Future research should also investigate comorbidities further as an additional explanation of the alcohol harm paradox and wider social inequalities.

## Data Availability

The datasets used in this study are available in the SAIL Databank at Swansea University, Swansea, UK, but as restrictions apply they are not publicly available. All proposals to use SAIL data are subject to review by an independent Information Governance Review Panel (IGRP). Before any data can be accessed, approval must be given by the IGRP. The IGRP gives careful consideration to each project to ensure proper and appropriate use of SAIL data. When access has been granted, it is gained through a privacy-protecting safe haven and remote access system referred to as the SAIL Gateway. SAIL has established an application process to be followed by anyone who would like to access data via SAIL at https://www.saildatabank.com/application-process .

## Appendix

**Table 6:** Sensitivity analysis using limited dataset following survey date only: comparison of model results for each socioeconomic measure

	Basic model	Adjusted model
	HR (95% CI; p-value)	HR (95% CI; p-value)
i1) Area deprivation (Model A/B)		
Less deprived 60% (ref)	1	1
Most deprived 40%	1.56 (0.93–2.59; 0.089)	1.27 (0.76–2.13; 0.367)
i2) Area deprivation and health (Model C)		
Less deprived 60% (ref)		1
Most deprived 40%		1.16 (0.69–1.96; 0.578)
i3) Area deprivation and mental health (Model D)		
Less deprived 60% (ref)		1
Most deprived 40%		1.25 (0.72–2.17; 0.429)
ii) Social class (NSSEC)		
Professional and managerial (ref)	1	1
Intermediate	1.7 (0.82–3.54; 0.155)	1.54 (0.74–3.21; 0.253)
Routine and manual	1.65 (0.9–3.04; 0.107)	1.38 (0.75–2.56; 0.302)
Never worked/long-term unempl.	6.08 (2.02–18.26; 0.001)	4.57 (1.50–13.94; 0.008)
iii) Employment		
Employed (ref)	1	1
Not employed	4.82 (1.73–13.41; 0.006)	3.89 (1.74–8.7; 0.001)
iv) Housing Tenure		
Home owner (ref)	1	1
Private rental	1.68 (0.68–4.17; 0.265)	1.55 (0.62–3.87; 0.346)
Social rental	4.9 (2.86–8.39; < 0.001)	3.84 (2.14-6.86; < 0.001)
V) Highest qualification		
Degree (ref)	1	1
Other	1.63 (0.69–3.87; 0.267)	1.17 (0.5–2.76; 0.719)
None	2.56 (1.02–6.45; 0.045)	1.71 (0.67–4.39; 0.264)

HR: hazard ratio; 95% CI: 95% confidence interval; N = 11,038; 60 events in less deprived, 71 in more deprived

## Abbreviations

95% CI: 95% confidence interval
ARHA: Alcohol-related hospital admission
BMI: Body Mass Index
ELAStiC: Electronic Longitudinal Alcohol Study in Communities
HR: Hazard ratio
LSOA: Lower layer Super Output Area
SAIL: Secure Anonymised Information Linkage
